# Tibia Fracture Healing Prediction Using First-Order Mathematical Model

**DOI:** 10.1155/2015/689035

**Published:** 2015-10-01

**Authors:** M. Sridevi, P. Prakasam, S. Kumaravel, P. Madhava Sarma

**Affiliations:** ^1^Saraswathy College of Engineering & Technology, Tindivanam, Tamil Nadu 604307, India; ^2^United Institute of Technology, Coimbatore, Tamil Nadu 641020, India; ^3^Thanjavur Medical College, Thanjavur, Tamil Nadu 613004, India

## Abstract

The prediction of healing period of a tibia fracture in humans across limb using first-order mathematical model is demonstrated. At present, fracture healing is diagnosed using X-rays. Recent studies have demonstrated electric stimulation as a diagnostic tool in fracture healing. A DC electric voltage of 0.7 V was applied across the fracture and stabilized with Teflon coated carbon rings and the data was recorded at different time intervals until the fracture heals. The experimental data fitted a first-order plus dead time zero model (FOPDTZ) that coincided with the mathematical model of electrical simulated tibia fracture limb. Fracture healing diagnosis was proposed using model parameter process gain. Current stabilization in terms of process gain parameter becoming constant indicates that the healing of fracture is a new finding in the work. An error analysis was performed and it was observed that the measured data correlated to the FOPDTZ model with an error of less than 2 percent. Prediction of fracture healing period was done by one of the identified model parameters, namely, process gain. Moreover, mathematically, it is justified that once the fracture is completely united there is no capacitance present across the fracture site, which is a novelty of the work.

## 1. Introduction

One of the major challenges in medical field is the prediction of fracture healing. The factors which decide nonunions of fractures in patients are the presence of unhealed fractured bone ends, viability of ends, and stability of the fixation and general bodily status of the individual which varies with presentation time and age of patient. Moreover, all the factors which control fracture healing are not known. The accepted theory of fracture healing is blood in fracture site becoming healed bone. The diagnosis of a bone fracture is confirmed and its union is tracked mainly with frequent X-rays, repeated at least during every clinical visit. Such frequent exposure to X-rays can cause marrow depression and malignancy [1–3]. Sometimes even around 30 X-rays are needed in a single patient till union [4]. X-ray analysis is not always consistent, as maturity in viewing a fracture radiograph depends on individual medical practitioner's skill and experience which leads to interobserver and intraobserver variations. Radiographic fracture healing assessments with fracture stiffness measurements were correlated [5] and study on biomechanical effects on bone to fully understand the mechanical properties of a patient's bone was illustrated [6]. Moreover, there is no agreement in defining what actually fracture union in an X-ray is [7, 8]. Few studies have tried enhancement of fracture healing with electrical stimulation [9, 10]. Recently, initial studies were carried out with a group of four tibial fractures cases with electric stimulation in the diagnostic side to monitor facture healing [11]. This was followed up with studies in an increased group size of 12 cases where authors have looked upon the current stabilization alone. When the current is stabilized, with the help of X-ray, they diagnose the fracture that was healed [12]. In an attempt to simplify the fracture healing process, models have been proposed to relate all possible data and observation to understand this process better. Some authors have proposed a first order system which has been tested and validated only on animals [13–15]. The General FOPDTZ (first order plus dead time and zero) model is represented as

(1) $$\begin{array}{c} G\left(s\right)=\frac{K_{p}Se^{-\tau_{d}S}}{\tau S+1}, \end{array}$$

where *K*_*p*_ is the process gain; *τ* is the process time constant; and *τ*_*d*_ is the measurement delay. In such process, when stabilization occurs, process gain is constant. To develop a model, steps to be followed are [16–18] collection of system data, identification of system, estimation of model parameter, and testing of the fitted model. The above authors have not analyzed fracture healing prediction by modeling using electrical data recorded across limb for humans. In this work, an alternative method to monitor fracture healing by simple mathematical model using electrical data recorded across human tibia fracture and its healing diagnosis using model parameter process gain has been proposed. Current stabilization in terms of process gain parameter becoming constant indicates the healing of fracture. This method was implemented to test fracture healing prediction for twelve patients at Thanjavur Medical College.

The paper is structured as follows.  Section 2 discusses methodology for modeling tibia fracture; Section 3 discusses development of mathematical model for tibia fracture.  Section 4 describes the experimental set-up for the fracture healing prediction.  Section 5 discusses validation of mathematical model through empirical model.  Section 6 deals with results and discussion, while Section 7 concludes this work.

## 2. Methodology for Modeling Tibia Fracture

**Figure 1:**
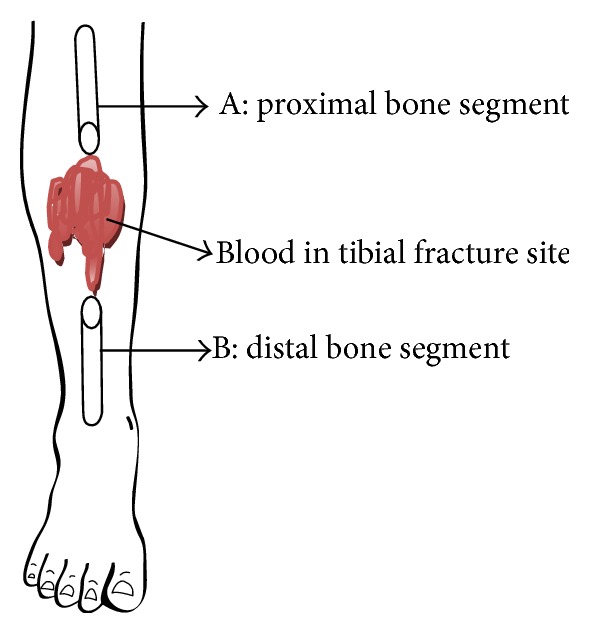
Broken bone.

When an intact bone is broken, there will be two pieces, for example, A and B, as shown in Figure 1. The gap between the two pieces will not be empty but filled with blood clot. If the electric current is passed from one end of the unbroken bone by an electrode, it reaches the electrode at the other end by the conduction property of an intact bone. If one attempts to test the same conduction through a fractured bone as in Figure 1, the current from the electrode passes through fragment A, then the blood clot, and then fragment B, before reaching the other electrode. The fracture site blood clot is considered as a dissimilar material between the two fractured fragments of bones A and B.

When a current is applied, this is considered as a dielectric and electrical conduction of a blood clot supported by the studies [19, 20] which is also realized in our present study by mathematical and empirical methods. Hence, we consider the tibia fracture site as a capacitance. Once the fracture site hematoma heals to become bone and becomes continuous with the two fragments A and B, the original conductivity and resistivity of an intact bone is restored to near normal. Once it was observed that the ionic transfer did not occur as evident from the asymptotic graphs, at this healed stage, the gain of the process is constant, which we found out in our model FOPDTZ (first order plus dead time zero) with constant gain.

In this study, 12-tibia fracture patients subjected to fracture healing by diagnostic DC simulation were studied. As a regular pattern of current, that is, initial irregularity in the current flow and its stabilization in later stage were observed in all the cases, modeling for four different fracture cases is demonstrated. Case 1 was an old patient; treatment was done on the day of the injury; that is, the ring was applied even before the infection could set in, within 6 hours of the injury and early union of the fractured bone was observed. The second case had first a debridement and a rod type external fixator. Later, it was converted to an interlocking nailing. Only when this nail got infected was he referred to our unit for Ilizarov ring fixation. The third case had first a debridement and a rod type external fixator. Later, this was removed as patient did not give consent to any further treatment. He later presented after 4 months to our unit for Ilizarov ring fixation. The second and third patients were middle aged who were treated after a minimum of two surgeries and hence had delay in healing. The fourth case had first a debridement and a rod type external fixator. He needed a plastic surgery in the form of flap cover from the calf muscle side. After settling of the flap, he needed repeated fracture site debridement causing loss of bone. He later presented after 5 months to our unit for Ilizarov ring fixation; the treatment procedure he had was a corticotomy and bone transport. The fourth case had a bone gap with prolonged treatment with bone transport. Remaining cases also rendered similar types of responses. The patient information for 12-tibia fracture trauma injuries is shown in Table 1.

## 3. Mathematical Modeling for Tibia Fracture

**Figure 2:**
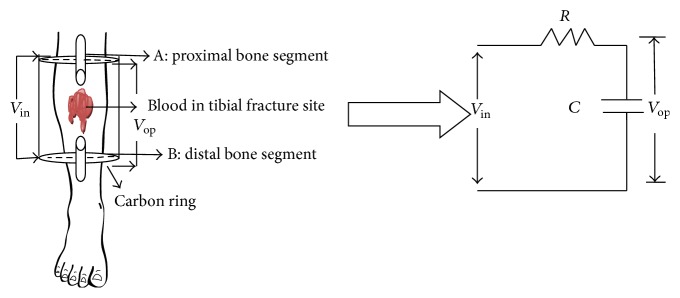
Equivalent circuit for single time constant model of tibia fracture.

As discussed before, the broken bone is considered as a capacitor; DC current is applied using with four Teflon coated carbon ring Ilizarov external fixators. The tibia fracture site acts as dielectric. Dielectric property of fracture site tissue changes with healing. The tibia fracture was analyzed, in modeling point of view, as two broken parts of bone with blood in between acting as capacitor [21]. The voltage to be applied to the capacitor (*c*) was passed in series through a resistor (*R*). The delay in recording the current in ammeter was taken as time delay (*τ*_*d*_). The equivalent circuit for single time constant model representation is shown in Figure 2.

The input to the single time constant model is the voltage *V*_in_. The output voltage is *V*_op_. *C* is the capacitance, *R* is the resistance, and *I* is the current flow into the system. Applying Kirchhoff's voltage law to the circuit shown in Figure 2, the following mathematical equation (1) was obtained:

(2) $$\begin{array}{c} V_{\text{in}}\left(t\right)=I\left(t\right)R+\frac{1}{c}\int I\left(t\right)dt. \end{array}$$

For stability analysis of nonlinear systems by applying Laplace transformation, we convert the parameters from time domain in (2) to parameters in “*S*” domain as represented in

(3) $$\begin{array}{c} V_{\text{in}}\left(s\right)=I\left(s\right)R+\frac{1}{CS}I\left(s\right), \end{array}$$

where *V*_in_(*s*) is input voltage, *I*(*s*) is the output current, and *S* is a complex variable, composed of real and imaginary parts: *S* = *σ* + *jω*, where *σ* is the real part and *jω* is the imaginary part. On rearranging, we obtain

(4) $$\begin{array}{c} V_{\text{in}}\left(s\right)=\frac{I\left(s\right)RCS+I\left(s\right)}{CS},\\ \frac{I\left(s\right)}{V_{\text{in}}\left(s\right)}=\frac{CS}{RCS+1}. \end{array}$$

Let *τ* = *RC*; *G*(*s*) = *I*(*s*)/*V*_in_(*s*); *K*_*P*_ = *C*; then the model is

(5) $$\begin{array}{c} G\left(s\right)=\frac{K_{P}S}{\tau S+1}. \end{array}$$

As all systems possess an inherent delay for the input to process through, process delay is introduced in (5) and the system with delay is represented in

(6) $$\begin{array}{c} G\left(s\right)=\frac{K_{p}Se^{-\tau_{d}S}}{\tau S+1}. \end{array}$$

In this tibia fracture, the time constant *τ* in the FOPDTZ model comprises the resistance as well as the capacitance of the fractured bone model whose values change as the broken bone heals. Thus, the time constant *τ* = *RC* changes too during fracture healing. The change in output current is thus due to changes in the bone's resistance and capacitance. This implies that the step response of the fractured bone changes as healing proceeds. This means that if we applied a step input (voltage) to the fractured bone, we would obtain a different step response every day. A constant voltage of 0.7 V was applied to the tibia fracture site and the current was recorded at various time intervals (days). The process gain *K*_*p*_ corresponds to change in output current to that of change in input voltage applied during treatment period (number of days). As the number of days increases, once the healing has started, the current drops down and becomes constant once the fracture has healed completely. Once the process gain becomes constant, we predict the healing of the fracture. From mathematical modeling of tibia fracture, it is clear that tibia fracture fits into FOPDTZ model.

## 4. Experimental Setup

**Figure 3:**
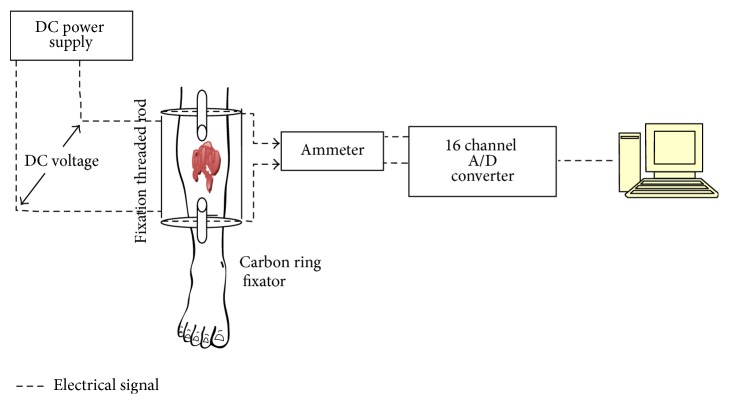
Experimental set-up for fracture healing model analysis.

The experimental set-up for fracture healing model analysis is shown in Figure 3. Data from the prospective study that was conducted where open fractures of tibia were treated was used in this study [15, 16]. The open fractures were cleaned of debris and contaminants and were stabilized with four Teflon coated carbon ring Ilizarov external fixators.

**Figure 4:**
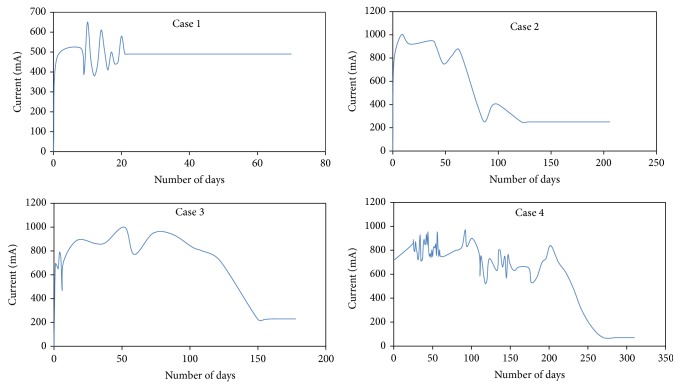
Experimental data collected from open loop response of four-tibia fracture patients' cases.

In these cases, the healing was followed with clinical assessment and periodical X-rays till the endpoint of fracture union and then the rings were removed. Additionally, all the patients also had application of electrical voltage in the range of 0.1–1.0 V DC in 0.1 V increments, across the two wires on either side of fracture. The output current was recorded by an ammeter connected in series. Ammeter measures the current flow across the fracture. Using the ammeter reading as reference, the online data recording of voltage calibration in terms of current is done. The schematic representation alone is shown in the experimental set-up. The wired diagram is published by one of the authors in [13–16]. The ammeter output was connected to M/s AD instrumentation 16-channel data acquisition card via signal conditioning unit. The card was connected to the USB port of the Pentium processor with an in-built antialiasing filter. The card supports 16 ADC and DAC channels in the range of ±15 V. Program was developed in “*C*” language to read and display the patient's current rating in terms of mA. The graph was compared with the appearance of new bone formation in X-rays. The above methodology was carried upon twelve different patients at Thanjavur Government Medical College to predict the exact instance at which a fracture has united completely. For all the twelve different patients, the same fracture healing pattern was obtained. The real-time experimental data for four tibia fracture patients is shown in Figure 4.

The initial irregularity of the graphs in Figure 4 constructed is already explained in certain papers [11, 12]. When skin wound healing was studied, there was stabilization of electrical potentials recorded across the wound after an initial irregularity as the skin wound recovered strength [13]. Moreover, bioelectric potentials after tibia fractures in rats stabilized after an initial period of irregularity [14]. Cellular and vascular processes in the early callus formation were cited to be the reason for the irregularity. The same concept is also applicable to fractured human bones in the referred study [12].

## 5. Empirical Model for Tibia Fracture

Using the experimental setup and electrical data obtained, empirical model was developed to predict the healing of fracture and error analysis was performed to validate the mathematical model. Model relies on input/output data for its training and capturing the dynamics of the process. In this study, the applied DC voltage is the input variable and the current across the tibia fracture is the output variable. A sampling time of 0.1 ms is used for the simulation. For the applied DC voltage, the resulting current values are stored in the MATLAB workspace. Here, the empirical model of the tibia fracture for CASE-1 is obtained by training the model with an input and output data of 1000 sets. Of these, 600 data pairs are used for training and the remaining 400 are used for validation of the network. As there was no change in the practical value, the data was limited to 1000 sets. Model Estimation Algorithm using Prediction Error Method in Matlab7.4 with system identification toolbox was written to estimate the FOPDTZ process model with one pole, one zero, gain, and time delay. The steps followed are as follows.

**Figure 5:**
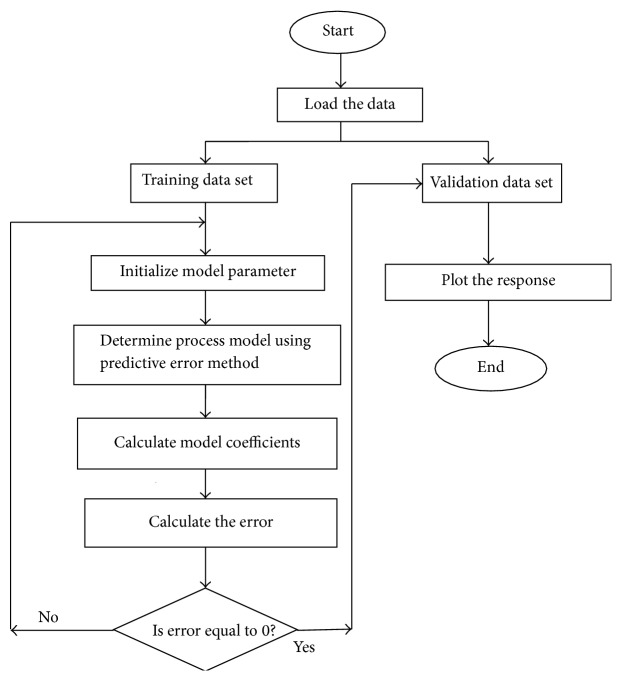
Process flow for estimating the model.

(1) Load the measured patient data individually for each patient.
(2) Classify the data into training data and validation data.
(3) Estimate the model and model coefficients using Prediction Error Method (PEM) for process models.
(4) Optimize the model to minimize sum of square of error *e*(*t*) difference between the measured output and the predicted output of the model.
(5) Perform the validation of model and plot the output response. The process flow for estimating the model is shown in Figure 5.

**Figure 6:**
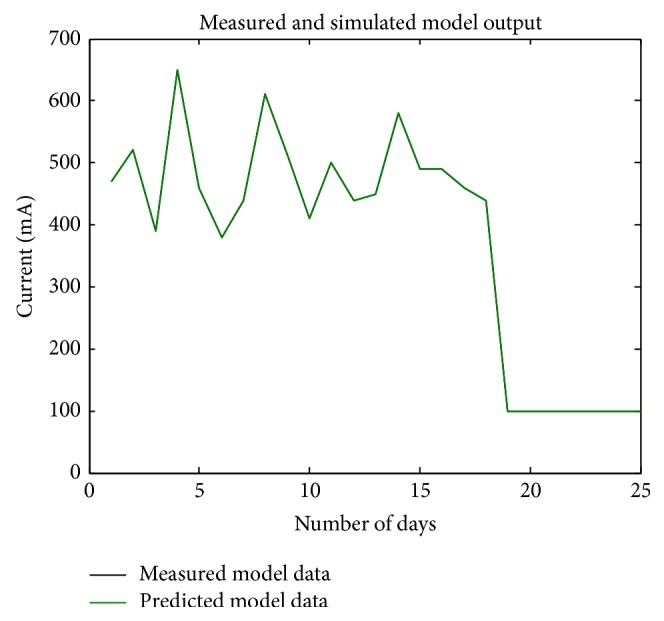
Comparison of measured and predicted output characteristics for tibia fracture modeled after introduction of zero (FOPDTZ).

**Figure 7:**
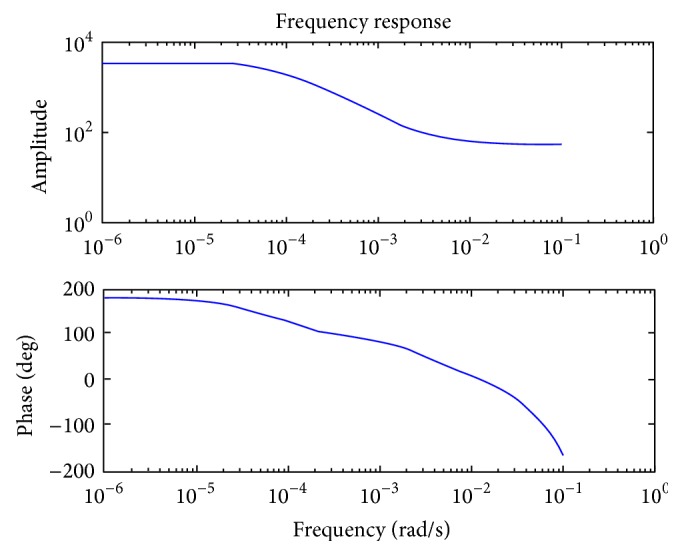
Frequency response characteristics for tibia fracture modeled after introduction of zero (FOPDTZ).

FOPDTZ model obtained using process model estimation technique [18] using MATLAB is shown in

(7) $$\begin{array}{c} G\left(s\right)=\frac{-3233.3\left(1-244.8s\right)e^{-30s}}{1+0.13846s}, \end{array}$$

where model parameters are process gain (*K*_*p*_) = −3233, time constant (*τ*) = 0.13846, and time delay (*τ*_*d*_) = 30. The empirical model obtained using electrical data is FOPDTZ model which is the same as that of the predicted mathematical model. Modeling was performed for the entire set of data as a whole recorded at different intervals rather than on day-to-day responses. The error analysis was performed for the FOPDTZ model shown in Figure 6. It was observed that measured and predicted data were identical and the average percentage error (APE) is zero. The system is able to predict the constant region due to introduction of zero in model. The frequency response characteristics are shown in Figure 7 and it was inferred from phase characteristics that there is no irregular phase shifts.

**Figure 8:**
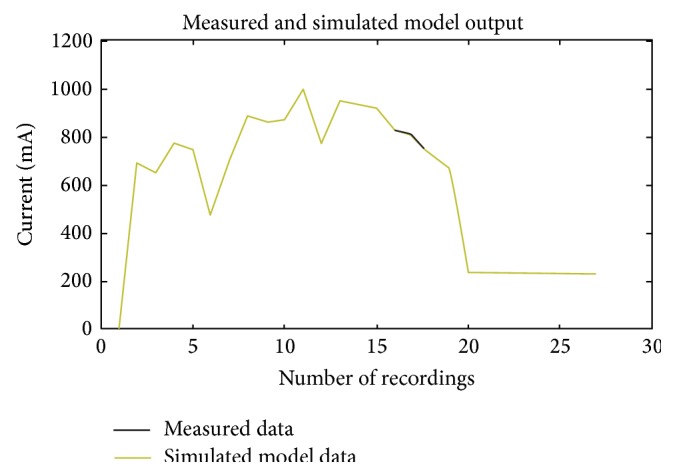
Measured and predicted output response for tibia fracture cases.

When the model was validated, it was observed that the average performance error (APE) was minimum. This experimental model coincided with the mathematical model of tibia fracture. The measured and predicted responses are shown in Figure 8. In Figures 6 and 8, the empirical model output when compared with actual experimental output was optimized to coincide so that we can visualize only one graph as both the measured and calculated output matched. However, we observed an average percentage error of 0.2 for case 4 as indicated in Table 2. The FOPDTZ model in (7) suits to mathematical model representation in (6). Hence, we can confirm that tibia fracture fits both mathematically and experimentally into FOPDTZ model.

## 6. Results and Discussion

Once the healing has been predicted, the rings are removed and ideal condition of capacitance becoming zero cannot be appreciated experimentally as there are no wires to apply the DC voltage. It has been inferred from the mathematical model that zero is needed for the system to match with actual measurement data. Hence, mathematically, we can justify that once the fracture is completely united there is no capacitance. Once the value of process gain *K*_*p*_ is constant, then the fracture has healed. The model validation is shown in Table 2. From Table 2, it is inferred that tibia fracture was modeled as FOPDTZ. The process gain was negative and it could be seen that the process gain becomes constant from Figure 8 around the 18th day. The X-ray was taken on the same day which also confirmed evidence of fracture healing.

It is hard, for example, in a human system, to try a hazardous maneuver of studying electrical conduction behaviors in uninjured limb. The mathematical models for all parts of the control system can be related together to simulate how the system would function under various disastrous maneuvers [15]. According to the type of the condition, models differ. In all these models, there is a main complexity in modeling a fracture healing process as there is certain void in the understanding of fracture healing process. On the other hand, if the main principles of such processes are only inadequately appreciated or if the mathematics of known principles is very intricate, then a physical model may be preferred, for example, New Zealand rabbits whose bones are osteotomized to simulate human fractures showed better union with electric stimulation compared to growth factors. Recent advance in computing has made mathematical modeling and the resulting graphic simulation progressively precise for different kinds of problems. The initial irregularity and later stabilization of current already reported were found well fitting in this model.

The effects of patients' factors on the values of parameters in the FOPDTZ model are described as follows. Various model parameters are process gain *K*_*p*_, time constant (*τ*), and time delay (*τ*_*d*_). Here, the time constant (*τ* = *RC*) mathematically relates to the capacitance of the tibia fracture site. We could observe from case 1, case 2, and case 3 that the values of time constant (*τ*) are 0.13, 0.003, and 16.4, respectively. Hence, mathematically, we can prove that the capacitance reaches zero by the value of *τ* on complete healing of process. When the value of *τ* is present, it gives an indication that the fracture gap still exists as observed in case 4. Moreover, when the process gain (which initially varies) finally becomes constant, it gives an indication that fracture has healed. The parameter time delay (*τ*_*d*_) indicates that the fracture reunion process is dependent on time. If multiple pieces of bone were present, then model can be developed using multiple capacitances connected in parallel to the resistor.

Here, in this study, we have considered open bone tibia fractures fixed with Illizario rings treated with DC electric stimulation where blood clot is present and considered as a capacitance. However, it would be interesting to know the applicability of the model to predict the healing process of stress fracture on tibia without any blood clot provided it is experimented on a patient who agreed for a ring fixator to understand the limitation of this study. We assume that, even in hairline fracture, there will be a minimal blood clot and callus formation for the fracture to heal.

## 7. Conclusion

FOPDTZ model was developed using the electrical data recorded across fractured limb that coincided with mathematical model. It was observed that the identified and measured data well fitted with minimum error for FOPDTZ model. The tibia fracture fitting into FOPDTZ model indicates that fracture tissue site capacitance becomes zero once it is completely healed. This fact is justified via modeling as it cannot be proved experimentally. Fracture healing is an intricate process with most of the parameters being unknown. Model will help to clarify the process and to study the effects of different components and to formulate predictions about fractures performance. We propose that when the process gain of the FOPDTZ model is constant the fracture has healed for the different tibia fracture cases which were also confirmed with X-ray diagnostic.

## Figures and Tables

**Table 1:** Patient information for 12 tibia fracture trauma injuries.

Name	Age (years)	Tibia fracture type	Site of fracture	Gender	General/specific health problems	Treatment	Date of stabilization	Date of ring removal	Number of X-rays
THY	37	Oblique	Middle third	Male	Normal	External fixator, split thickness skin grafting, and interlocking nailing	137	158	10

SMK	55	Oblique	Middle third	Male	Diabetes mellitus	External fixator	91	134	8

ADR	25	GAP	Middle third	Male	Normal	External fixator, muscle flap to cover bone	292	355	34

VSW	39	GAP	Middle third	Male	Ischemic Heart Disease	External fixator, split thickness skin grafting	189	243	20

TMLS	17	Short oblique	Lower third	Male	Normal	Plaster slab immobilization	110	187	8

KND	50	Short oblique	Lower third	Male	Normal	Plaster slab immobilization	56	133	12

JAYS	29	Open comminuted with bone loss	Middle third	Male	Normal	External Fixator	85	120	12

RVN	30	Oblique	Upper third	Male	Normal	Plaster slab immobilization	106	127	4

VDVL	30	Oblique	Middle third	Male	Normal	Plaster slab immobilization	67	82	6

RJSK	28	Open comminuted with bone loss	Lower third	Male	Normal	Calcaneal pin traction to maintain alignment of the fracture	160	159	10

STMR	28	Oblique	Middle third	Male	Normal	External fixator	67	84	6

**Table 2:** Model validation of the tibia fracture model.

Patient	Process model	Order	Pole	Zero	Model parameters					APE (%)
					*K*_*p*_	*τ*	τ_1_	τ_2_	τ_*d*_	
Case 1	$\frac{\begin{array}{c} -3233.3(1-244.8s)e^{-30s} \end{array}}{1+0.13846s}$	First	1	1	−3233	0.13846	0	0	30	0

Case 2	$\frac{-2868(1+6e^{5}s)e^{-29s}}{1+0.003s}$	First	1	1	−2868	0.003	0	0	29	0

Case 3	$\frac{-3.8e^{12}(1+4.2e^{8}s)e^{-30s}}{\left(1+16.4s\right)}$	First	1	1	−3.8*e* ^12^	16.4	0	0	−30	0

Case 4	$\frac{-5.8e^{6}(1+2.6s)e^{-30s}}{\left(1+17e^{6}s\right)}$	First	1	1	−5.8*e* ^6^	17*e* ^6^	0	0	30	0.2
